# A152 INTESTINAL INFLAMMATION DISRUPTS MICROBIAL ANTIGEN METABOLISM FACILITATING FOOD SENSITIZATION

**DOI:** 10.1093/jcag/gwae059.152

**Published:** 2025-02-10

**Authors:** B Barbosa da Luz, L E Rondeau, P Muppidi, C Ling, R Dang, A Caminero

**Affiliations:** McMaster University, Hamilton, ON, Canada; McMaster University, Hamilton, ON, Canada; McMaster University, Hamilton, ON, Canada; McMaster University, Hamilton, ON, Canada; McMaster University, Hamilton, ON, Canada; McMaster University, Hamilton, ON, Canada

## Abstract

**Background:**

Patients with inflammatory bowel disease (IBD) report diverse food-related adverse reactions. Dairy is the most self-reported food intolerance in IBD. Emerging evidence suggests that altered diet-microbiota interactions can contribute to the development of IBD and adverse reaction to foods. We hypothesize that microbial alterations in IBD patients facilitate adverse reactions to foods.

**Aims:**

To investigate the role of intestinal inflammation on microbial metabolism and food sensitization.

**Methods:**

To investigate the effects of inflammation on microbial metabolism of food, eight-week-old C57BL6 mice were exposed to different concentrations (1.5 – 3.5%) of dextran sodium sulfate (DSS) for 5 days, then gavaged with dairy proteins. To study the role of inflammation in food sensitization, C57BL/6 mice were exposed to 3% DSS for 5 days, then sensitized to dairy proteins (6 mg casein/whey protein) using 4 oral administrations of cholera toxin (20 ug/mouse). After 1 week of recovery, sensitized mice were exposed to either a dairy-free or dairy containing diet for 7 days. Finally, the effects of inflammatory-mediated microbial alterations on food sensitivities were studied in gnotobiotic mice. Germ-free C57BL6 were colonized with DSS-inflamed microbiota or healthy SPF microbiota. After 3 weeks of colonization, the mice were sensitized to dairy proteins as described above, exposed to dairy and one cycle of DSS. Intestinal microbiota composition (16S sequencing) and function (digestion assays), and markers of intestinal inflammation (disease activity index, histological damage, cell infiltration and pro-inflammatory genes expression) were analyzed.

**Results:**

DSS led to pronounced changes in the intestinal microbiota, impaired microbial capacity to degrade dairy proteins and increased systemic access of antigens. Inflammation also facilitated food sensitization characterized by immune cell infiltration (polymorphonuclear cells and CD3^+^) and mast cell proliferation in the colonic mucosa, and generation of systemic IgE and IgG levels. Dairy increased genes associated to immune cell recruitment and apoptosis in sensitized mice, and increased of apoptosis in the colon was confirmed by staining (TUNEL). Finally, mice colonized with microbiota from DSS-inflamed mice showed a worsening in inflammatory parameters during the dairy-diet intervention after sensitization. These results suggest that microbial alterations after inflammation facilitate the onset of food sensitivities and exacerbate food-mediated immune reactions in the colon.

**Conclusions:**

Intestinal inflammation facilitates sensitization to foods by altering microbial antigen metabolism in the colon.

**Funding Agencies:**

CCC

